# Supplementary material for: The Role and Regulation of Autophagy and the Proteasome During Aging and Senescence in Plants
Source: Genes (Basel). 2019 Apr 2;10(4):267. doi: 10.3390/genes10040267 (PMC6523301; doi:10.3390/genes10040267)
Supplement: Supplementary file 1 [file genes-10-00267-s001.pdf]

Table S1. Detected post-translational modifications (PTMs) on proteasome subunits of *Arabidopsis*.

Subunits	Gene-ID	Quantity	Types, Locations of PTMs
**Rpt1a**	AT1G53750	6	V^{2}nt, K^{13}ub, S^{96}ph, T^{125}ph, C^{370}ro, K^{400}ub
**Rpt1b**	AT1G53780	2	S^{289}ph, C^{563}ro
**Rpt2a**	AT4G29040	3	G^{2}my, H^{123}nt, K^{353}nt
**Rpt2b**	AT2G20140	3	G^{2}my, H^{123}nt, K^{353}nt
**Rpt3**	AT5G58290	7	A^{2}na/nt, S^{16}ph, A^{18}nt, K^{70}ac, C^{369}no, K^{387}ac
**Rpt4a**	AT5G43010	6	T^{2}nt/na, K^{48}ac, S^{80}ph, K^{203}ub, K^{304}nt
**Rpt4b**	AT1G45000	4	K^{48}ac, S^{80}ph, K^{203}ub, K^{304}nt
**Rpt5a**	AT3G05530	7	A^{2}na/nt, C^{50}no, S^{52}ph, C^{225}ro, T^{294}nt, N^{300}nt
**Rpt5b**	AT1G09100	3	A^{2}na/nt, C^{224}ro
**Rpt6a**	AT5G19990	4	A^{2}na/nt, C^{19}ro/no
**Rpt6b**	AT5G20000	2	A^{2}na/nt
**Rpn1a**	AT2G20580	8	S^{40}ph, M^{107}mo, K^{113}ac, K^{168}ac, K^{218}ub, T^{343}ph, S^{359}ph, C^{621}no
**Rpn1b**	AT4G28470	3	M^{72}mo, T^{343}ph, S^{359}ph
**Rpn2a**	AT1G08410	0	
**Rpn2b**	AT2G32730	11	A^{2}na, K^{279}ac, T^{318}nt, S^{412}nt, A^{433}nt, K^{730}ac, S$^{876/878/891/896}$ph, K^{903}nt
**Rpn10**	AT4G38630	12	V^{2}nt, K^{130}ac, S^{136}ph, S^{187}nt, T^{188}nt, D^{258}nt, S$^{263/265/363}$ph, K$^{379/380/386}$ub

Subunits	Gene-ID	Quantity	Types, Locations of PTMs
**Rpn3a**	AT1G20200	9	T^{2}nt, T^{12}ph, S$^{14/16/19/20}$ph, T$^{22/24}$ph, C^{240}no
**Rpn3b**	AT1G75990	8	T$^{12/14}$ph, S$^{16/19}$ph, T^{22}ph, S^{23}ph, T^{24}ph, C^{239}no
**Rpn5a**	AT5G09900	6	G^{2}na/nt, S^{4}ph, K^{19}ub, C^{234}no, K^{320}ac
**Rpn5b**	AT5G64760	2	K^{19}ub, C^{234}no
**Rpn6**	AT1G29150	5	V^{2}na/nt, C$^{51/332}$no, M^{364}mo
**Rpn7**	AT4G24820	1	M^{1}na
**Rpn8a**	AT5G05780	3	M^{1}na, S^{218}ph, Y^{219}ph
**Rpn8b**	AT3G11270	1	M^{1}na
**Rpn9a**	AT5G45620	1	A^{2}na
**Rpn9b**	AT4G19006	3	A^{2}na/nt, A^{3}na
**Rpn11**	AT5G23540	2	K^{238}ub, S^{265}ph
**Rpn12a**	AT1G64520	2	M^{1}na, C^{190}no
**Rpn12b**	AT5G42040	1	C^{156}no

**PAA1**	AT5G35590	2	K$^{33/181}$ac
**PAA2**	AT2G05840	1	K^{181}ac
**PAB1**	AT1G16470	5	G^{2}na, K^{50}ac, K^{92}ub, K$^{165/171}$ac
**PAB2**	AT1G79210	5	G^{2}na, K^{50}ac, K^{92}ub, K^{165}ac, K^{171}ac
**PAC1**	AT3G22110	8	K^{40}ub, K^{198}ac, M^{200}mo, T^{218}ph, S$^{220/232}$ph, T^{234}ph, k^{239}ac
**PAC2**	AT4G15160	0	
**PAD1**	AT3G51260	3	K^{29}ub, L^{99}nt, K^{176}ac
**PAD2**	AT5G66140	3	K^{29}ub, L^{99}nt, K^{176}ac
**PAE1**	AT1G53850	3	K^{52}ac, K^{66}ub, E^{95}na
**PAE2**	AT3G14290	4	K^{52}ac, K$^{66/185}$ub, E^{95}na
**PAF1**	AT5G42790	7	M^{1}na, K^{51}ub, S^{90}ph/nt, S^{92}ph, k^{169}ub/ac
**PAF2**	AT1G47250	7	M^{1}na, K^{51}ub, S^{90}ph/nt, S^{92}ph, K^{169}ub/ac
**PAG1**	AT2G27020	6	S^{2}ph/na/nt, K$^{173/240}$ac, M^{246}mo
**PBA1**	AT4G31300	4	T^{13}nt, S^{58}ph/nt, S^{60}ph
**PBB1**	AT3G27430	4	T^{40}nt, G^{50}nt, G^{84}nt, A^{85}nt
**PBB2**	AT5G40580	4	T^{40}nt, G^{50}nt, G^{84}nt, A^{85}nt
**PBE2**	AT3G26340	5	K$^{47/53}$ub, T^{58}nt, K$^{89/128}$ac
**PBF1**	AT3G60820	2	V^{115}nt, S^{163}ph
**PBD2**	AT4G14800	0	

**PBD1**	AT3G22630	1	M^{36}mo
**PBC1**	AT1G21720	2	S^{2}na, K^{98}ac
**PBE1**	AT1G13060	2	$K^{89/128}$ac
**PBC2**	AT1G77440	2	S^{2}na, K^{98}ac
**PBG1**	AT1G56450	2	$S^{234/235}$ph
**UMP1-like**	AT1G67250	0	
**UMP1-like**	AT5G38650.1	0	

Illustrated example: V^{2}nt, V: modified valine; 2: second amino acid of protein sequence; nt: PTM types. Abbreviation of different PTMs: ac/lysine acetylation, bu/lysine 2-hydroxyisobutyrylation, ca/carbonylation, ma/lysine malonylation, me/lysine methylation, mo/methionine oxidation, my/myristoylation, na/N-terminal acetylation, ng/N-glycosylation, no/S-nitrosylation, nt/N-terminus proteolysis, nu/N-terminal ubiquitination, og/O-GlcNAcylation, ph/phosphorylation, ro/reversible cysteine oxidation, sg/S-glutathionylation, sm/lysine SUMOylation, su/lysine succinylation, ub/lysine ubiquitination.
